# A Metagenomics Investigation of Intergenerational Effects of Non-nutritive Sweeteners on Gut Microbiome

**DOI:** 10.3389/fnut.2021.795848

**Published:** 2022-01-14

**Authors:** Weilan Wang, Jodi E. Nettleton, Michael G. Gänzle, Raylene A. Reimer

**Affiliations:** ^1^Faculty of Kinesiology, University of Calgary, Calgary, AB, Canada; ^2^IWK Health Centre, Division of Gastroenterology and Nutrition, Halifax, NS, Canada; ^3^Department of Agricultural, Food and Nutritional Science, University of Alberta, Edmonton, AB, Canada; ^4^Department of Biochemistry and Molecular Biology, Cumming School of Medicine, University of Calgary, Calgary, AB, Canada

**Keywords:** non-nutritive sweeteners, metagenomic construction, gut microbiome, maternal diet, obesity risk

## Abstract

To identify possible mechanisms by which maternal consumption of non-nutritive sweeteners increases obesity risk in offspring, we reconstructed the major alterations in the cecal microbiome of 3-week-old offspring of obese dams consuming high fat/sucrose (HFS) diet with or without aspartame (5–7 mg/kg/day) or stevia (2–3 mg/kg/day) by shotgun metagenomic sequencing (*n* = 36). High throughput 16S rRNA gene sequencing (*n* = 105) was performed for dams, 3- and 18-week-old offspring. Maternal consumption of sweeteners altered cecal microbial composition and metabolism of propionate/lactate in their offspring. Offspring daily body weight gain, liver weight and body fat were positively correlated to the relative abundance of key microbes and enzymes involved in succinate/propionate production while negatively correlated to that of lactose degradation and lactate production. The altered propionate/lactate production in the cecum of weanlings from aspartame and stevia consuming dams implicates an altered ratio of dietary carbohydrate digestion, mainly lactose, in the small intestine vs. microbial fermentation in the large intestine. The reconstructed microbiome alterations could explain increased offspring body weight and body fat. This study demonstrates that intense sweet tastants have a lasting and intergenerational effect on gut microbiota, microbial metabolites and host health.

## Introduction

Low-calorie sweeteners, also known as non-nutritive sweeteners, have been used for decades to replace sugar and reduce the caloric content of foods and beverages while maintaining the sweet taste (1). The consumption of non-nutritive sweeteners has raised concerns about potential detrimental effects of long-term intake which have been demonstrated in some rodent studies (2–5) but less consistently in human studies (4, 6–8). Aspartame and stevia are two commonly used low-calorie sweeteners whose metabolism has been investigated in different species (3, 9–14). Aspartame is a dipeptide-methyl ester (4) which, upon ingestion, is rapidly metabolized into methanol, aspartate and phenylalanine and absorbed into the systemic circulation (4, 13). Rebaudioside A, the stevia glycoside used in this study, is resistant to hydrolysis by pancreatic or brush border enzymes but is converted to the aglycone steviol by ileal or colonic bacteria expressing β-glucosidases; absorption and metabolism of steviol in the liver occurs in a similar manner in humans and rats (11, 12, 15). *Bacteroides* isolates from human microbiota were identified as the most efficient bacteria in hydrolyzing rebaudioside A to steviol (12).

Non-nutritive sweeteners are non-toxic to human adults (3, 9, 11) but detrimental effects, including disrupted gut microbiota, impaired glucose homeostasis and higher risk of obesity have been observed in offspring of rodents consuming non-nutritive sweeteners and infants of pregnant women consuming beverages with non-nutritive sweeteners (2, 3, 5, 7, 9). We recently reported that maternal consumption of aspartame and stevia altered the expression of genes related to the mesolimbic reward system in 3-week and 18-week old rat offspring, and altered gut microbiota in the 3-week old offspring (9). Both changes could plausibly explain the associations between maternal aspartame and stevia consumption with the higher body weight gain and body fat of their offspring (9). The former could promote higher food intake while the latter is related to the energy derived from intestinal absorption of nutrients and microbial metabolites. At the same time, we transplanted fecal microbiota of the offspring from aspartame and stevia consuming dams to germ free mice, which resulted in an increase in glucose intolerance, body weight gain and body fat of the recipient mice (9). Despite the compositional changes detected in this study and the convincing transfer of the obese phenotype into germ free mice through fecal microbiota transplant, our understanding of the functional changes that occurred in the offspring microbiome in response to maternal consumption of low-calorie sweeteners remains limited.

Therefore, the objective of the present study was to reconstruct the major alterations in the cecal microbiome of 3-week old offspring that are related to maternal consumption of aspartame or stevia during pregnancy and lactation. Short chain fatty acids (SCFA), the end products of gut microbial fermentation, were selected as the most direct mediator to investigate microbial-associated energy metabolism (16). We use shotgun metagenomics and 16S rRNA gene sequencing to elucidate the connections between key microbes, metabolic functions, and the physiological outcomes of offspring, with the goal of identifying the possible mechanisms by which maternal aspartame and stevia consumption exert effects on offspring that never directly consumed the sweeteners themselves.

## Materials and Methods

### Animals and Samples

**Figure 1:**
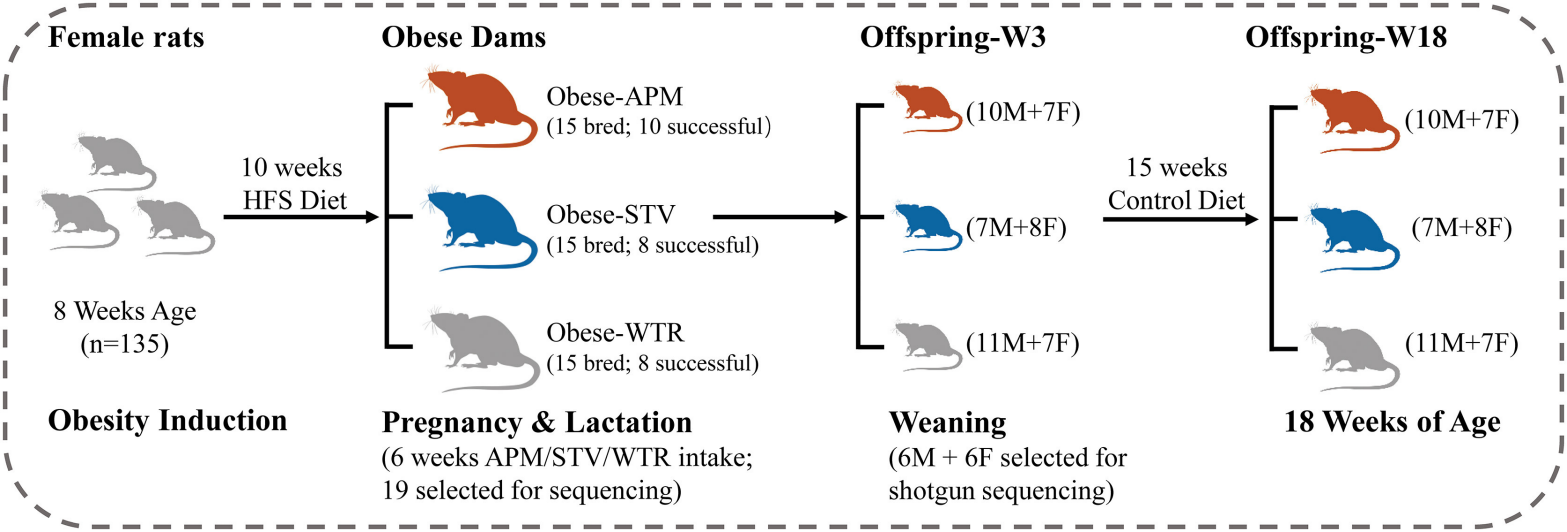
Study design. HFS, high fat/sucrose diet; APM, aspartame; STV, stevia; WTR, water control; M, male; F, female; W, week.

Ethical approval was granted by the University of Calgary Animal Care Committee (Protocol#AC15-0079) and conformed to the *Guide to the Care and Use of Experimental Animals*. Obesity was induced in 8-week-old female Sprague-Dawley rats using a 10-week feeding period with a high fat/high sucrose diet (HFS, 39% fat and 44% sucrose, Dyets #102412), the composition of which has been previously published (17). Given that Sprague-Dawley rats can be obesity-prone or obesity-resistant when fed a HFS diet (18), we chose the top *n* = 45 best weight gainers from the *n* = 135 rats included in the diet-induced obesity protocol (Figure 1). The selected obese female rats were bred with male Sprague-Dawley rats and randomly allocated to one of three groups of *n* = 15 each throughout pregnancy and lactation: (1) WTR (HFS + Water); (2) APM (HFS + aspartame) (5–7 mg/kg, Fluka, Ottawa, ON, Canada); (3) STV (HFS + stevia) (2–3 mg/kg Rebaudioside A, Sigma-Aldrich, Oakville, ON, Canada). Aspartame and stevia were administered in the drinking water as described (9). These doses were selected within the acceptable daily intake (aspartame: 40 mg/kg, stevia: 4 mg/kg) (19) established by the Food Directorate of Health Canada and reflect physiological daily intake in humans (20). To minimize the effects of varying litter size, litters were culled to 10 offspring (5 females + 5 males) at birth. Offspring were weaned at 3 weeks of age and fed a control diet (AIN-93, Dyets, Bethlehem, PA, USA) until 18 weeks of age. Rats were fed *ad libitum* and housed in a temperature- and humidity-controlled room with a 12-h light/dark cycle. Body weight was measured weekly. Body composition of the dams was measured at weaning and the offspring measured at 3 and 18 weeks of age using dual-energy X-ray absorptiometry (DXA) (Hologic QDR 4500; Hologic Inc., Marlborough, MA, USA). Each litter was considered as *n* = 1 and male and female offspring were housed separately in plastic cages with wood-chip bedding following weaning at 3 weeks of age.

Distal jejunum and ileum tissue samples, and cecal digesta were collected at terminate points of dams (at weaning) and offspring (at 3 and 18 weeks of age) and immediately stored at −80°C until use. Total bacterial DNA was isolated from cecal digesta using FastDNA spin kit for feces (MP Biomedicals, Lachine, QC, Canada), pre-treated with bead-beating (MP Biomedicals, Lachine, QC, Canada) for 40 s × 3 times. Purified DNA was quantified using Quant-iT™ PicoGreen™ dsDNA Assay Kit (Invitrogen, Burlington, ON, Canada) and diluted to 20 ng/μl for 16S rRNA gene sequencing and 50 ng/μl for shotgun metagenomic sequencing.

### Analysis of Cecal Microbiota by 16S rRNA Gene Sequencing

To assess the contributions of maternal and offspring factors to cecal microbiota variations, genomic DNA from 105 samples were randomly selected for 16S rRNA gene sequencing, corresponding to 19 dams at weaning, 36 offspring at 3 weeks of age, and 50 offspring at 18 weeks of age. Microbial 16S rRNA gene tags were sequenced on Illumina MiSeq platform (2 × 300 bp) by amplifying the hypervariable V3–V4 regions (Centre for Health Genomics and Informatics, Calgary, AB, Canada), and analyzed in QIIME2 platform (21) (QIIME2 2020.2). The quality controlled 16S rRNA were filtered and denoised using DADA2 in QIIME2 (21) (QIIME2 2020.2). Frequency of sequence variants less than 10 were discarded. Taxonomy was assigned to sequence variants using Silva 138 database as reference. Sequence variants classified as genus *Lactobacillus* were additionally aligned to Genome Taxonomy Database release 95 (GTDB, https://gtdb.ecogenomic.org/) to reflect the current taxonomy of *Lactobacillaceae* (22) with the best BLAST hits.

### Recovery of Bacterial Genomes From Shotgun Metagenomic Reads

To reconstruct cecal microbiome of offspring, 36 genomic DNAs from offspring at 3 weeks of age (weaning) were randomly selected for shotgun sequencing that included 12 samples (6 female and 6 males) from each dietary group. Shotgun metagenomic sequencing was conducted on NovaSeq 6000 (2 × 300 bp, Centre for Health Genomics and Informatics, Calgary, AB, Canada) with NEB Ultra II library preparation protocol (New England Biolabs, Ipswich, MA, USA) to generate 400 GB of data (800 M reads). Due to the high percentage of the unidentified rodent microbiome and the high functional heterogeneity in microbial community at the strain level, we adopted *de novo* assembly-based metagenomic reconstruction to understand the complexity of functional alterations in offspring's gut microbiota (23, 24). Adapters were checked and removed by Trimmomatic (25). Quality-controlled reads were assembled into contigs/scaffolds by samples using default parameters in IDBA_UD (26). Long contigs (> 3,000 bp) were further clustered into bins using MaxBin2 (27) based on G + C content and abundance of contigs through Expectation-Maximization algorithm. The 16S rRNA gene sequences data and shotgun metagenomic sequences data generated during this study are available at the National Center for Biotechnology Information (BioProject PRJNA675294) https://www.ncbi.nlm.nih.gov/biosample.

### Taxonomic Position and Relative Abundance of Metagenomic Assembled Genomes

In total, 188 representative bins were chosen by de-replicating highly similar genomes using dRep (28), which refers to metagenomic assembled genomes (MAGs). Genomes completeness and contamination were estimated by examining a set of ubiquitous genes using CheckM (29) (Supplementary Table 1).

Phylogenetic trees of MAGs were built with PhyloPhlAn 3.0 (30) by aligning to a set of 400 core proteins. Species-, Genus- and Family-level taxonomic labels were assigned to genomes when Mash (31) average genomic distance calculated from at least 100 makers was < 5%, 15% and 30% to the closest reference set, respectively (30). The phylogenetic tree was generated with Randomized Axelerated Maximum Likelihood (RAxML) method and visualized by iTOL (32). Raw reads were mapped back to MAGs to calculate relative abundance using CoverM with “relative_abundance” formula in “genome” model.

### Evaluation of Microbial Metabolism Potentials by Genome-Wide Screening

To estimate the contribution of the cecal microbiome to microbial-derived energy harvest, metabolic enzymes were selected to retrieve pathways for microbial degradation of lactose and production of lactate, succinate, acetate, propionate, and butyrate (Supplementary Table 2). Query sequences for the same enzyme were selected from different bacteria and pre-aligned to minimize the overlap between BLAST hits for the same enzyme. Amino acid sequences of biochemically characterized key enzymes were blasted against amino acid database build from the open reading frames (ORF) of 188 assembled genomes (Prodigal 2.0) (33). An alignment coverage of > 70%, *e* value ≤ 1e-5 and amino acid identity of ≥40% or higher (34) were used as cut-off values for BLAST. The sums of mapped reads (%) for all the positive hits for the same enzymes were calculated as their potential capacities to produce the corresponding metabolites in each sample.

### RNA Extraction and Quantification of Gene Expression

Total RNA was extracted from jejunum and ileum tissue using Trizol (Invitrogen, Carlsbad, CA) and reverse-transcribed into cDNA using SuperScriptII RT as previously described (35). Quantitative PCR was performed on BioRad iCycler (Bio-Rad, Hercules, USA) with primer sets LCT (forward, 5′-AATCTTCTTGGCTGGGAATGG-3′; reverse, 5′-CCTTGAGCACCTCGTTGA TG-3′) and GATA4 (forward, 5′-AATCTTCTTGGCTGGGAATGG-3′; reverse, 5′-CCTTGA GCACCTCGTTGATG-3′) targeted at lactase gene (*lct*) and regulatory gene (*gata4*). The 18S gene was amplified as reference gene for relative quantification with primer set (forward, 5′-TGACTCAACACGGGAAACC-3′; reverse, 5′-TCGCTCCACCAACTAAGAAC-3′). The relative expression ratio (R) of targets were calculated based on primer efficiency (E) and the Ct deviations as follow: R = (${{\text{(E}}_{\text{target}})}^{\text{Δ Ct target }(\text{control-sample})}$ / (E_ref_)^Δ Ct ref (control−sample)^ (36).

### Statistical Analysis

The data for 16S rRNA gene sequences and shotgun metagenomic sequences, including relative abundance of 16S rRNA gene sequence variants, weighted UniFrac distances, relative abundance of assembled genomes and the corresponding prediction of metabolite production were compared using Kruskal-Wallis rank-sum test in R version 4.0.0 (2020-04-24). Statistical significance of ANOSIM was determined through 999 permutations between dietary groups. The R value between 0 and 1 reflects the dissimilarity between the groups and was calculated as follows: R = difference of mean rank (all distances between groups – all distances within groups) / (N(N-1)/4). The data for host parameters, including daily weight gain, body fat (%), liver weight, and bone mineral density, and Log_10_ transformed gene expression were analyzed using linear mixed-effects (LME) models in R version 4.0.0 0 (2020-04-24). Dietary treatment was treated as fixed factor; rat was considered as experimental unit and its random effect was removed. Correlations between genome, metabolite production and host parameters were analyzed by Spearman's rank method. Bonferroni-adjusted *p* < 0.05 were considered significant.

## Results

### Maternal Consumption of Aspartame and Stevia Influences Gut Microbiota of Offspring

**Figure 2:**
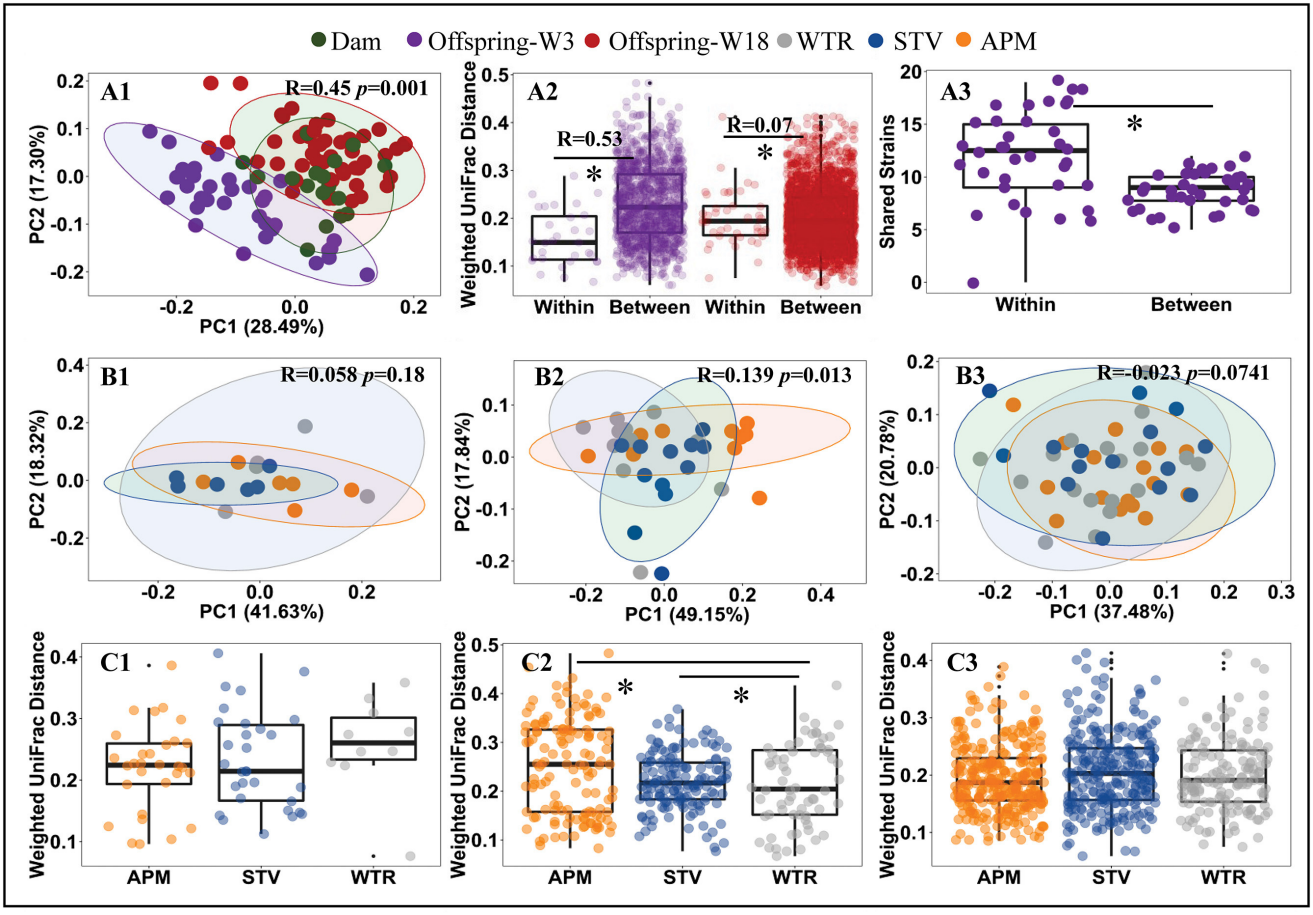
Influence of maternal consumption of aspartame and stevia on cecal microbiota in rat dams and their offspring. Principle coordinate analysis (PCoA) **(A1, B1–B3)** of rat cecal microbiota; litter effects in offspring **(A2)**; shared metagenomic assembled genomes **(A3)** between 3-week-old offspring from the same litter (left) and from different litters (right); Dietary effects in dams **(C1)**, 3-week-old offspring **(C2)**, and 18-week-old offspring **(C3)**. Each dot represents an individual cecal sample. Data with asterisk (*) represents a significant difference (*p* < 0.05). W3, week 3; W18, week 18; APM, aspartame; STV, stevia; WTR, water control.

Consumption of low-dose aspartame and stevia showed limited influence on the overall structure of cecal microbiota in dams (Figures 2A1,B1,C1; Supplementary Table 3) but significantly altered (*p* < 0.001) cecal microbiota of their 3-week old offspring (Figures 2A2,B2,C2; Supplementary Table 3). The differences at 3 weeks gradually dissipated from weaning into adulthood (Figures 2A2,B3,C3; Supplementary Table 3). Dams and 18-week-old offspring shared more similarities in cecal microbiota (Figure 2A1; Supplementary Table 3). Significant litter effects (male and female from same dam) were observed in 3-week old offspring, as indicated by the weighted UniFrac distances (Figure 2A2, R = 0.53, *p* = 0.001) and a higher number of highly similar (>99.0% average nucleotide identity) bacterial genomes that were shared between offspring from the same dam (Figure 2A3, *p* < 0.001). Litter effects decreased in 18-week-old offspring (Figure 2A2, R = 0.07, *p* = 0.0741). No sex difference was observed in 3-week and 18-week (*p* > 0.05) old offspring. Therefore, subsequent metagenomic analyses focused on microbiome alterations in 3-week old offspring without stratification by sex.

### Metagenomic Reconstruction of Cecal Microbiome in 3-Week Old Rats

**Figure 3:**
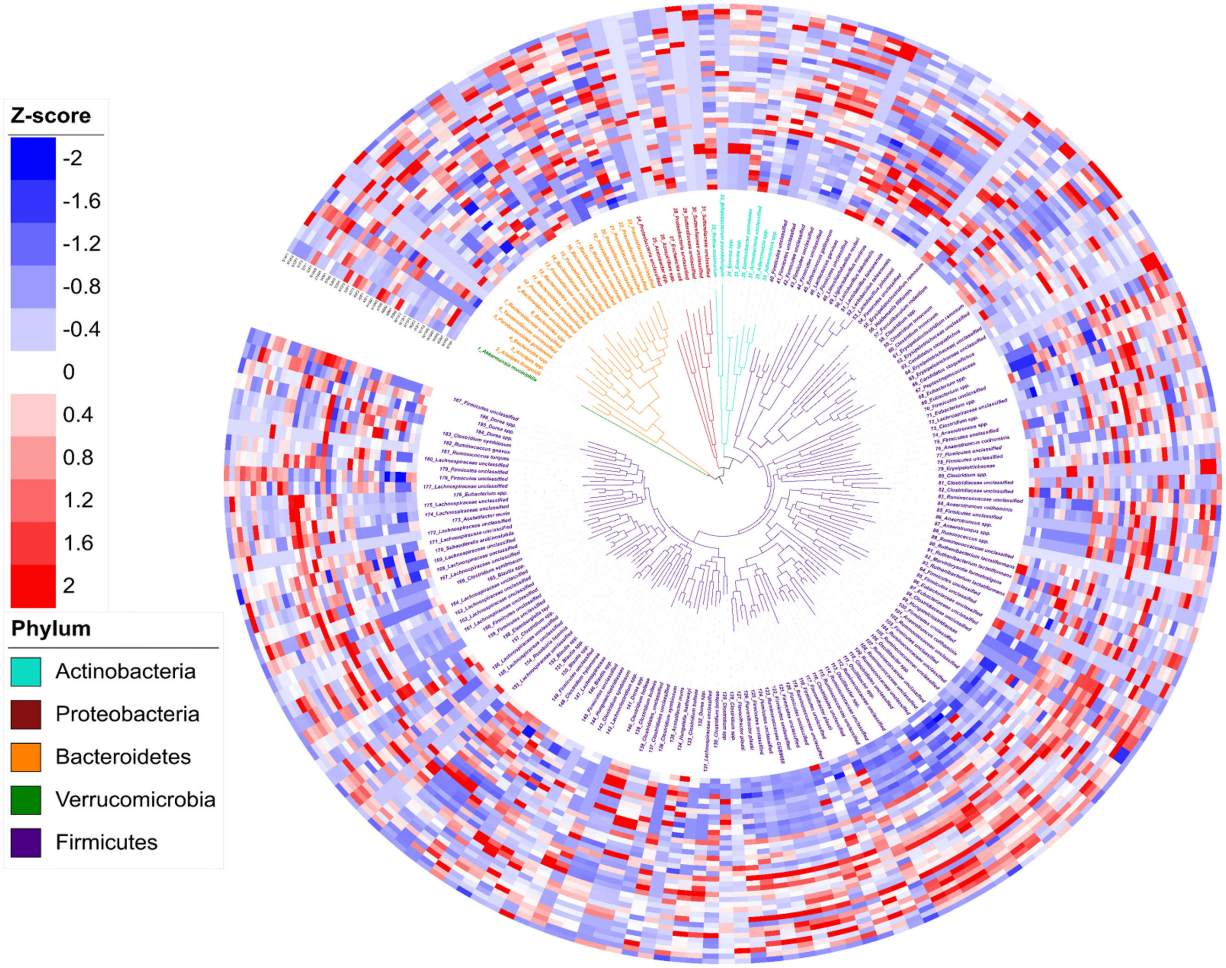
Phylogeny and abundance of metagenomic assembled genomes recovered from 3-week old rat cecal digesta. The relative abundance of bins was calculated from percentage of re-mapped reads in the sample and presented as Z-score: Z-score = (relative abundance – mean of relative abundance)/standard deviation. Phylogenetic tree (the innermost layer) and taxonomic affiliations (the middle layer) of 188 metagenomic assembled genomes (MAGs). Tree branches and labels with different colors represent different phyla as indicated by the color code in the lower left. The outermost heatmap depicts the relative abundance of the 188 bins in each sample, inside to outside: Water Control (12 layers), Aspartame (12 layers) and Stevia (12 layers).

**Figure 4:**
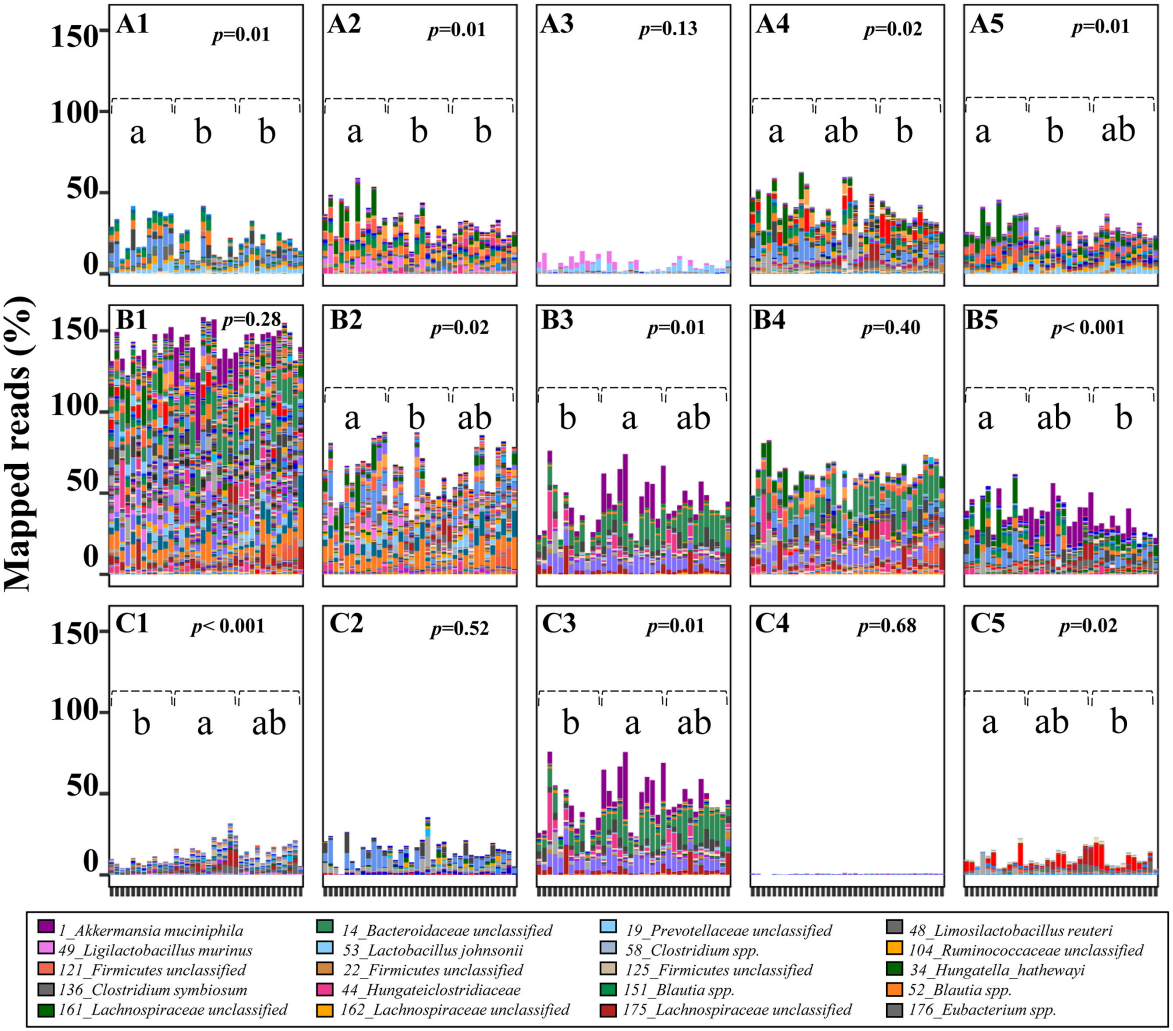
Predicted potential of cecal microbiome involved in lactose and SCFA metabolism in 3-week old offspring. The potential of each cecal microbial community to degrade lactose and generate SCFA-related metabolites was calculated by the sum of mapped reads (%) for positive hits of the corresponding key enzymes as follow: Lactose **(A1,A2)** beta-galactosidase (LacZ and Lacdeb), **(A3–A5)** phospho-β-galactosidase (LacG, BbgI and LacM); Acetate **(B1)** acetate kinase (AK); Lactate **(B2)** D/L-lactate dehydrogenase (LacDeh); Succinate, **(B3)** fumarate reductase (frdA); 1,2-Propanediol; **(B4)**, lactaldehyde reductase (LacRd); Cobalamin (Vitamin B12), **(B5)** cobalt transporter protein (CbiM); Butyrate **(C1)** butyryl:acetate CoA-transferase (BCoA), **(C2)** butyrate kinase (BK); Propionate, **(C3)** methylmalonyl-CoA decarboxylase (McoA), **(C4)** lactoyl-CoA dehydratase (LacCoRd), **(C5)** propanediol dehydratase (pduCDE). Data with different superscripts represents significant difference (*p* < 0.05). For bars in each panel, left to right: water control (*n* = 6); aspartame (*n* = 6) and stevia (*n* = 6).

In total, 188 genomic bins were recovered from 36 3-week old offspring cecal samples, with an average size of 3.23 Mb and an average N50 of 74,759 bp (Supplementary Table 1). The high quality of reconstructed bacterial genomes enabled genome-based analysis of metabolic traits of intestinal microbial communities. Figure 3 depicts the phylogeny of 188 metagenomic assembled genomes. Five bacterial phyla were assigned to these 188 assembled genomes, including 1 assigned to *Verrucomicrobia*, 7 identified as *Actinobacteria*, 10 as *Proteobacteria*, 23 assigned to *Bacteroidetes*, and 137 identified as *Firmicutes*. Of the 188 assembled genomes, 149 (79%) were assigned to genus or species level with an average amino acid identity of > 95% to the closest references calculated from at least 100 protein sequences. The relative abundance of each genome, referring to the percentage of mapped reads, was further transferred to Z-scores for visualization (Figure 4). In total, 92 genomes showed differential relative abundance (*p* < 0.05) between dietary treatments (Supplementary Figure 1).

### Reconstruction of Cecal SCFA Production Related Metabolic Pathways in 3-Week Old Rats

To estimate the contribution of the cecal microbiome to microbial-derived energy harvest, pathways for microbial degradation of lactose and for production of lactate, succinate, acetate, propionate, and butyrate were reconstructed (Figure 4; Supplementary Table 1). Intracellular GH2 β-galactosidases BbgI, LacM, LacZ and Lacdeb, specifically distributed in *Lachnospiraceae, Lactobacillaceae* and *Clostridiales* were determined as the major metabolic enzymes hydrolysing milk-derived lactose in rats (Figures 4A1–A5). The relative abundance of these β-galactosidases significantly decreased in the stevia and/or aspartame groups compared to water control (Figures 4A1,A2,A4,A5, *p* < 0.05). Phospho-β-galactosidase LacG (Figure 4A3) showed low abundance and no differences between sweeteners and water control groups in the cecal microbiota. The abundance of enzymes for pyruvate to lactate conversion (Figure 4B) was significantly reduced (*p* < 0.05) in the aspartame group compared to the stevia and water control.

Rat cecal microbes (90.43%) were generally able to produce acetate through acetate kinase (AK) (not shown) and due to the multiple mapping of identical reads, the average of mapped read ratio for acetate was higher than 130%. Variable bacterial types and abundance were involved in acetate production, but no difference was observed in total abundance between dietary groups (Figure 4A). Very few bacteria produce both propionate and butyrate as major products of carbohydrate fermentation (Supplementary Figure 1). *Firmicutes* genomes were identified as the major butyrate producers. Of these butyrate producers, 15 of 28 butyryl: acetate CoA-transferase carrying genomes and 10 of 17 butyrate kinase carrying genomes were significantly enriched in both aspartame and stevia groups or specifically enriched in aspartame (*p* < 0.05). Of the three pathways for propionate production, the succinate pathway, dominated by *Akkermansia muciniphila* and *14_Bacteroidaceae* unclassified, was the most abundant (30 of 46 propionate-producing genomes). This pathway was significantly enriched in the aspartame group. Of the 30 succinate-propionate producers, 9 genomes were enriched by aspartame and stevia or only by aspartame (4 of 9). The propanediol pathway for propionate production was less abundant in the stevia group although the production of 1,2-propanediol was not different between dietary groups. Genes coding for the acrylate pathway were rare in the rat cecal microbiome. Production of cobalamin (vitamin B12), a co-factor required by multiple metabolic processes including propionate production, also showed variable abundances in aspartame and/or stevia offspring.

### Reducing Dimensions of Microbial Population Differences and Their Correlations With Host Growth and Body Composition

**Figure 5:**
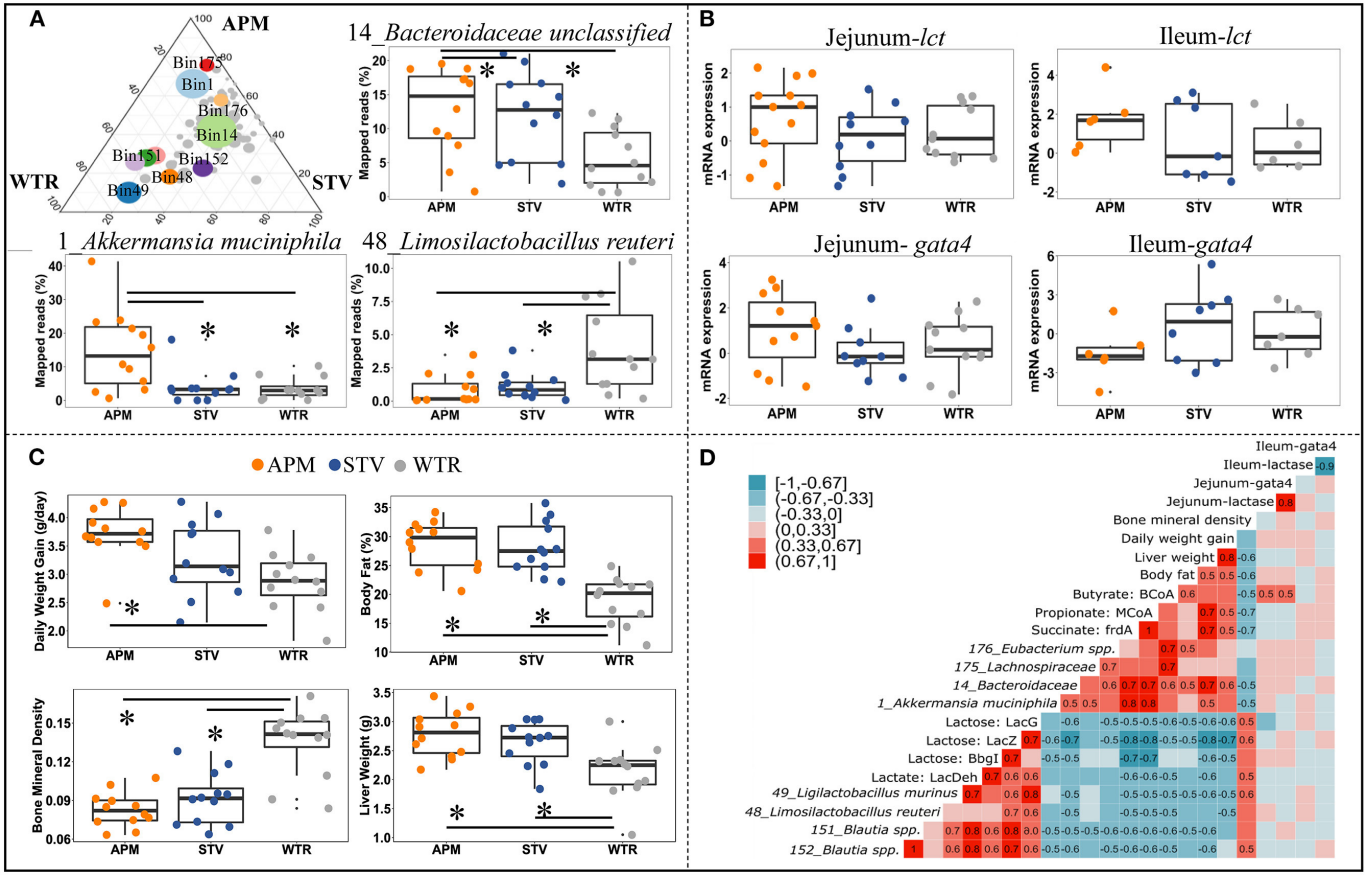
Connections between cecal microbiome alterations and host parameters in 3-week old offspring. The top 10 influenced genomes in cecal microbiota of 3-week-old offspring (ternary plot) and the relative abundance of 3 representative influenced genomes (boxplots) **(A)**; mRNA expression fold change of jejunal/ileal lactase gene (*lct*) and GATA4 (*gata4*) **(B)**; Growth peformance and body composition of weanlings **(C)**; The coefficient matrix of Spearman's rank correlations between the most influenced genomes, metabolic enzymes, gene expression, and the corresponding growth and body composition of 3-week old offspring **(D)**. Data with asterisk (*) represents significant difference (*p* < 0.05). APM, aspartame; STV, stevia; WTR, water control.

To further explore the influence of non-nutritive sweeteners on the cecal microbiome of offspring, genomes with high relative abundance and significant differences between groups were selected as representatives for further correlation analysis (Figure 5A; Supplementary Table 4). The genome of *14_Bacteroidaceae* unclassified represented the most abundant microbe that was enriched in both aspartame and stevia groups; the genome of *1_Akkermansia muciniphila* represented bacteria specifically enriched by aspartame; microbes reduced by aspartame were represented by *48_Limosilactobacillus reuteri* and *49_Ligilactobacillus murinus*. The other genomes, including *176_Eubacterium* spp., *151_Blautia* spp*., 152_Blautia* spp., and *175_Lachnospiraceae* unclassified, showed variable differences between dietary treatments. Jejunal and ileal expression of lactase and its regulator GATA4 can directly affect the availability of lactose for fermentation in the cecum. The expression of these genes was therefore examined as a potential upstream factor shaping the microbiome, however no significant differences between dietary treatments were found (Figure 5B). Host parameters, including daily weight gain, body fat, liver weight, and bone mineral density were selected for correlation analysis (Figure 5C). Maternal consumption of aspartame increased daily weight gain in offspring (Figure 5C). Aspartame and stevia significantly increased body fat % and liver weight but decreased bone mineral density in 3-week old offspring (Figure 5C).

Correlations between bacterial genomes, bacterial metabolites, and between host parameters depict microbial-host interactions in weanling pups (Figure 5D). Two distinct bacterial consortia were identified, showing negative correlations with each other and consistently opposite correlations with metabolite production and host parameters. The propionate- and butyrate-producing consortium, represented by *1_Akkermansia muciniphila, 14_Bacteroidaceae* unclassified, *175_Lachnospiraceae* unclassified, and *176_Eubacterium* spp., was enriched in offspring of sweetener-consuming dams, and was positively correlated with offspring's daily body weight gain, liver weight and body fat but negatively correlated with bone mineral density (Figure 5D). Conversely, the lactate-producing consortium, including *48_Limosilactobacillus reuteri, 49_Ligilactobacillus murinus, 151*_*Blautia spp*., and *152*_*Blautia spp*., which degrade lactose into lactate, were negatively correlated to the host parameters mentioned above except bone mineral density (Figure 5D). Significant correlations between gene expression of lactase/GATA4 gene and selected bacterial genomes and host parameters were only observed between jejunal *lct*/*gata4* with butyrate formation (Figure 5D).

## Discussion

In this study, we demonstrate that the altered intestinal microbiota resulting from consumption of non-nutritive sweeteners by dams explains the negative metabolic changes occurring in their offspring that were themselves never directly exposed to the sweeteners. To identify possible mechanisms by which this intergenerational risk is transmitted, we assessed the metagenomic reconstruction of intestinal metabolism of dietary carbohydrates in the offspring alongside host parameters including weight gain, body fat, liver weight and bone mineral density. We show that maternal consumption of aspartame or stevia altered microbial metabolism in the offspring; specifically, the relative abundance of propionate- and butyrate-producing species increased, and the abundance of lactose-fermenting species decreased in offspring at weaning. The resultant altered propionate and lactate production could explain increased body weight and body fat in offspring from aspartame and stevia consuming dams. Importantly, we show that sweet tastants have a lasting and intergenerational effect on gut microbiota, microbial metabolites and host health.

Artificial sweeteners induce compositional and functional alterations in gut microbiota of human and rats (5, 8, 9). These shifts in gut microbiota have been linked to the development of obesity-related glucose intolerance which is transferable to germ-free mice through fecal microbiota transplantation (5, 9, 37). Maternal dietary intake during gestation and lactation is known to program offspring health *via* multiple mechanisms (37–39), which include transmission of gut microbiota and its metabolic potential (37, 40). We examined litter effects on microbiota structural similarities and shared identical bacterial genomes between offspring from the same dam vs. different dams and confirmed that maternal microbial transmission plays an important role in the development of cecal microbiota of 3-week old offspring.

Given the obese phenotype of offspring derived from dams consuming non-nutritive sweeteners, we focused our metagenomics reconstruction on the pathways for the production of acetate, propionate, and butyrate. We observed increased readouts of succinate/propionate conversion and butyrate formation in the cecal microbiome of offspring from aspartame and stevia consuming dams and their positive correlation with increased liver weight, body weight gain, and body fat. Altered SCFA metabolism, especially elevated serum and fecal propionate, has been shown previously in obese animals following artificial sweetener consumption (3, 5, 8, 9). Exogenous administration of propionate to healthy individuals in a meal resulted in hyperglycemia which was primarily attributed to an increase in serum glucagon and overexpression of fatty acid-binding protein4 (5). Beyond higher blood glucose and hyperinsulinemia, significantly higher body weight gain and body fat was also observed in mice that were administered a chronic low dose of propionate in their drinking water compared to control mice (41). These findings are in line with the impaired glucose tolerance we observed in male offspring of our aspartame dams (9). We also observed increased mRNA levels of dopamine transporter in the ventral tegmental area of the male weanlings of sweetener consuming dams (9). Upregulation of the dopamine transporter could imply an activation of the mesolimbic reward pathway in the brain that promotes food-seeking behavior, particularly to highly palatable foods (9). In depth analysis of central and systemic appetite regulation in offspring of sweetener consuming dams, including the satiety hormones, PYY and GLP-1, are warranted to better understand the influence of altered microbial metabolism on appetite regulation in these animals (41, 42).

Understanding how shifts in endogenous propionate production affect the development of metabolic abnormalities, however, remains challenging. We show that maternal intake of aspartame and stevia and the resultant early colonizers that are transmitted from dams impacted the cecal microbiome of offspring. Host diet and particularly the content of indigestible but fermentable carbohydrate plays a key role in shaping the gut microbiome (43). Milk-derived lactose accounts for 12–15% of rat milk solids during late lactation (44, 45). In human infants and suckling rats, a substantial proportion of dietary lactose escapes small intestinal hydrolysis and absorption and is fermented in the large intestine (46, 47). The cecal/colonic lactate that is produced from lactose fermentation in turn serves as a substrate for butyrate and propionate production by other members of the intestinal microbiota (48–50). Therefore, we extended our pathway construction to lactose/lactate metabolism and identified the reduced proportions of metagenomic reads mapped to lactose hydrolase and lactate formation, and their negative correlations with propionate/butyrate production and weight gain and body fat in offspring from dams that consumed non-nutritive sweeteners. Notably, host glycans, mainly mucin, are degraded predominantly by *Akkermansia* and *Bacteroides* species (51, 52). In line with the highly enriched glycan degradation pathways found in mice consuming saccharin (5), the increased relative abundance of *Akkermansia* and *Bacteroidetes* with a concomitant lower proportion of lactobacilli in our study also suggests that maternal consumption of non-nutritive sweeteners increases the relevance of host glycans but decreases the relevance of lactose as substrate for cecal carbohydrate fermentation in their offspring. Decreased lactate formation from lactose by lactobacilli results in an increased cecal pH, which further benefits the mucin-degrading *Akkermansia* and *Bacteroidetes*(5, 53, 54) and shifts cecal microbial metabolism (34, 50).

More research is needed to evaluate the possible role of non-nutritive sweeteners on offspring metabolism *via* their presence in breastmilk. Important to the current investigation is that while some low-calorie sweeteners have been detected in breastmilk (i.e., saccharin, sucralose, and acesulfame-potassium), this does not appear to be true for all sweeteners including aspartame (6). In addition, the concentration of non-nutritive sweeteners in breast milk after maternal consumption, even if detectable, is too low to impact gut microbiota through direct provision of any substrate and these sweeteners are not known to alter lactose content of milk or host glycans secretions (2, 6, 15, 55). Given the impact of jejunal and ileal lactase activity on the availability of lactose for intestinal microbiota in suckling rats and the decline in lactase activity which is essentially regulated at the posttranscriptional level during the late lactation period (56–59), we examined the gene expression of jejunal/ileal lactase and regulator GATA4 but did not find a difference between offspring of dams consuming non-nutritive sweeteners or not. Although we cannot rule out other possibilities, the lack of difference reduces the likelihood that lactose metabolism had an upstream effect on cecal SCFA production and suggests that obesity risk in offspring after maternal consumption of non-nutritive sweeteners is mediated by the host response to an altered pattern of bacterial metabolites. The possibility remains that exposure to altered bacterial metabolites that have previously been detected in the maternal fecal and/or serum metabolome when mothers consume sucralose and aspartame (3, 60), could contribute.

## Conclusions

Our metagenomic reconstruction of cecal SCFAs metabolism identified decreased lactose fermentation and an altered propionate and lactate production as the central tenet of increased body weight and body fat in offspring from aspartame and stevia consuming dams. Although the mechanisms of transmission of this phenotype from mother to offspring and its perpetuation into adulthood remain to be elucidated, this study demonstrates the intergenerational effect that sweet tastants have on gut microbiota, microbial metabolites and host health. This result has important implications for human health because the diet of mothers during pregnancy and lactation likely also impacts the gut microbiota, microbial metabolites, and the metabolic fitness to their children. Compositional and functional shifts in the microbiome with non-nutritive sweetener consumption should be investigated further in human cohorts to inform guidelines for maternal diet during pregnancy.

## Data Availability Statement

The datasets presented in this study can be found in online repositories. The names of the repository/repositories and accession number(s) can be found below: https://www.ncbi.nlm.nih.gov/, BioProject PRJNA675294.

## Ethics Statement

Ethical approval was granted by the University of Calgary Animal Care Committee (Protocol#AC15-0079).

## Author Contributions

WW designed research, prepared samples, performed bioinformatics analysis, generated figures, and wrote paper. JN designed and performed original animal experiments and reviewed the paper. MG assisted with analysis and interpretation and reviewed the paper. RR designed research, assisted with interpretation, obtained funding, and had primary responsibility for final content. All authors have read and approved the final manuscript.

## Funding

This work was supported by the Natural Sciences and Engineering Research Council of Canada through grant RGPIN-2016-03773. WW is supported by a University of Calgary Eyes High Postdoctoral Fellowship. JN was supported by Alberta Children's Hospital Research Institute and Canadian Institutes of Health Research. MG is supported by the Canada Research Chairs Program.

## Conflict of Interest

The authors declare that the research was conducted in the absence of any commercial or financial relationships that could be construed as a potential conflict of interest.

## Publisher's Note

All claims expressed in this article are solely those of the authors and do not necessarily represent those of their affiliated organizations, or those of the publisher, the editors and the reviewers. Any product that may be evaluated in this article, or claim that may be made by its manufacturer, is not guaranteed or endorsed by the publisher.
