# Supplementary material for: A Metagenomics Investigation of Intergenerational Effects of Non-nutritive Sweeteners on Gut Microbiome
Source: Front Nutr. 2022 Jan 14;8:795848. doi: 10.3389/fnut.2021.795848 (PMC8794796; doi:10.3389/fnut.2021.795848)
Supplement: Supplementary file 1 [file Data_Sheet_1.PDF]

Supplementary Material

1 Supplementary Figures and Tables

1.1 Supplementary Tables

Supplementary Table 1. Quality assessment and taxonomy of 188 metagenomic assembled bins

**Bin_ID**	**Taxonomy**	**Score**	**Completeness (%)**	**Contamination (%)**	**Size (bp)**	**N50**
S1_Maxbin_out.037	*137_Clostridiales unclassified*	94.98	95.92	0.68	3138035	83464
S1_Maxbin_out.058	*15_Prevotellaceae unclassified*	72.34	84.99	3.2	4499634	8975
S1_Maxbin_out.061	*47_Firmicutes unclassified*	7.17	96.55	18.49	4262131	56420
S10_Maxbin_out.008	*16_Bacteroidaceae unclassified*	95.25	95.69	0.57	3481701	64796
S10_Maxbin_out.009	*104_Ruminococcaceae unclassified*	94.55	97.86	1.36	2981344	143601
S10_Maxbin_out.024	*163_Lachnospiraceae unclassified*	96.03	93.6	0	2749370	71681
S10_Maxbin_out.025	*146_Blautia spp.*	86.28	98.85	3.07	6613710	370671
S10_Maxbin_out.026	*19_Prevotellaceae unclassified*	98.30	96.09	0	2879776	26475
S10_Maxbin_out.036	*130_Clostridium bolteae*	65.65	95.08	6.38	3904782	85588
S10_Maxbin_out.040	*119_Ruminococcaceae unclassified*	35.81	79.4	9.18	2731008	12627
S10_Maxbin_out.054	*69_Eubacterium spp.*	28.73	95.3	14	3929749	24860
S10_Maxbin_out.057	*168_Lachnospiraceae unclassified*	-25.15	79.72	21.55	3139955	2580
S11_Maxbin_out.011	*105_Ruminococcaceae unclassified*	98.92	97.99	0.34	2299130	183617
S11_Maxbin_out.021	*40_Firmicutes unclassified*	95.59	96.79	0.75	2174237	128104
S11_Maxbin_out.032	*173_Acetatifactor muris*	70.91	98.66	6.04	2854837	77942
S11_Maxbin_out.036	*55_Erysipelatoclostridium ramosum*	-1.60	86.23	18.65	3916241	7616
S11_Maxbin_out.038	*141_Dorea spp.*	-4.85	88.01	19.75	2759629	10348
S11_Maxbin_out.040	*43_Firmicutes unclassified*	47.15	87.27	8.46	3205958	4416
S11_Maxbin_out.043	*39_Adlercreutzia spp.*	6.27	96.49	19.65	3533497	68516
S12_Maxbin_out.018	*154_Roseburia hominis*	96.46	93.96	0	1855746	99792
S12_Maxbin_out.026	*117_Flavonifractor plautii*	99.62	99.28	0.48	3291761	295705
S12_Maxbin_out.028	*11_Bacteroidetes unclassified*	93.71	94.63	0.67	3378876	71786
S12_Maxbin_out.040	*183_Clostridium symbiosum*	-2.92	90.45	19.1	2723523	17791
S12_Maxbin_out.062	*46_Lactococcus garvieae*	-36.49	83.15	24.26	1660715	2101
S13_Maxbin_out.019	*122_Firmicutes unclassified*	88.07	91.43	1.13	2335621	38664
S13_Maxbin_out.029	*25_Acetobacter spp.*	99.55	99.06	0.38	3035789	59771
S14_Maxbin_out.001	*147_Lachnospiraceae*	101.92	99.33	0	3270092	152733
S14_Maxbin_out.011	*184_Dorea spp.*	99.67	97.26	0	2657000	67581
S14_Maxbin_out.019	*177_Lachnospiraceae unclassified*	60.04	85.51	5.64	3485558	66729
S14_Maxbin_out.020	*144_Hungateiclostridiaceae*	90.02	97.7	2.01	4433585	53984
S14_Maxbin_out.028	*102_Ruminococcaceae unclassified*	51.94	98.25	9.82	4493395	49086
S15_Maxbin_out.028	*109_Ruminococcaceae unclassified*	95.51	97.88	0.96	3322757	72928

S16_Maxbin_out.003	*27_Escherichia coli*	98.30	100	0.81	3673839	49850
S16_Maxbin_out.016	*36_Gordonibacter pamelaeae*	93.57	97.38	1.25	2358862	75532
S17_Maxbin_out.005	*49_Lactobacillus murinus*	96.07	100	1.27	3570743	67709
S18_Maxbin_out.001	*60_Clostridium innocuum*	101.92	99.43	0	4289885	96628
S18_Maxbin_out.002	*14_Bacteroidaceae unclassified*	97.06	99.62	1.03	6635127	154634
S18_Maxbin_out.024	*113_Oscillibacter spp.*	56.74	78.14	4.64	2937994	3920
S18_Maxbin_out.048	*4_Bacteroides spp.*	86.22	94.62	2.15	1905618	49012
S19_Maxbin_out.005	*134_Hungatella_hathewayi*	87.86	95.48	2.01	4735535	72225
S19_Maxbin_out.007	*139_Clostridium bolteae*	101.27	98.64	0	3482725	183323
S19_Maxbin_out.021	*28_Proteobacteria unclassified*	102.31	100	0	2920408	42260
S19_Maxbin_out.024	*136_Clostridium symbiosum*	99.38	98.87	0.38	2728182	66443
S19_Maxbin_out.048	*37_Actinobacteria unclassified*	32.11	89.54	12.01	2240108	9431
S2_Maxbin_out.008	*90_Ruthenibacterium lactatiformans*	101.31	98.91	0	2122498	64384
S20_Maxbin_out.010	*35_Enorma spp.*	98.12	98.75	0.62	2111968	87544
S20_Maxbin_out.012	*151_Blautia spp.*	101.77	99.37	0	3699733	64263
S20_Maxbin_out.023	*80_Clostridium spp.*	19.10	96.87	16.05	2717553	14214
S20_Maxbin_out.048	*93_Ruthenibacterium lactatiformans*	20.20	96.87	15.88	4196938	37135
S21_Maxbin_out.008	*58_Clostridium spp.*	102.50	100	0	2985421	99349
S21_Maxbin_out.020	*138_Clostridiales_unclassified*	97.47	97.04	0.38	3181447	44914
S21_Maxbin_out.023	*107_Ruminococcaceae unclassified*	84.55	97.47	3.07	4076820	73583
S21_Maxbin_out.025	*17_Bacteroidaceae unclassified*	101.37	98.68	0	2752003	236393
S21_Maxbin_out.030	*91_Ruthenibacterium lactatiformans*	93.98	98.58	1.42	2259526	102172
S21_Maxbin_out.031	*54_Firmicutes unclassified*	85.89	90.49	1.41	2205052	9211
S21_Maxbin_out.032	*31_Sutterellaceae unclassified*	98.34	95.97	0	2803838	55079
S21_Maxbin_out.043	*100_Firmicutes unclassified*	51.17	76.78	6.59	1940527	13928
S21_Maxbin_out.046	*143_Clostridium symbiosum*	2.40	93.86	20.41	3160848	16097
S22_Maxbin_out.017	*61_Erysipelatoclostridium ramosum*	101.21	99.62	0.21	4723256	193957
S23_Maxbin_out.018	*145_Firmicutes unclassified*	87.76	96.14	2.35	2308902	24193
S23_Maxbin_out.020	*180_Lachnospiraceae unclassified*	88.03	89.13	0.67	2406768	31313
S23_Maxbin_out.023	*92_Marvinbryantia formatexigens*	101.76	99.36	0	5939093	63916
S23_Maxbin_out.036	*56_Holdemania filiformis*	90.23	94	1.26	2756696	113705
S24_Maxbin_out.014	*81_Clostridiaceae unclassified*	94.01	99.33	1.74	6828435	237447
S24_Maxbin_out.031	*148_Clostridium hylemonae*	97.47	96.5	0.32	3098187	140066
S24_Maxbin_out.033	*62_Erysipelotrichaceae unclassified*	99.95	97.32	0	2392554	178457
S24_Maxbin_out.043	*127_Flavonifractor plautii*	33.70	87.81	11.28	3245878	38159
S24_Maxbin_out.045	*45_Enterococcus gallinarum*	60.94	85.82	5.48	2914051	12835
S25_Maxbin_out.008	*152_Blautia spp.*	98.28	98.08	0.48	2452825	159063
S25_Maxbin_out.009	*157_Clostridium spp.*	80.17	81.02	0.73	2084103	44109
S25_Maxbin_out.028	*128_Clostridium spp.*	97.43	98.58	0.71	2988648	62889
S25_Maxbin_out.031	*42_Firmicutesunclassified*	66.67	99.11	7.06	4991463	56746
S26_Maxbin_out.013	*123_Ruminococcaceae*	94.83	99.12	1.34	4916520	65685
S26_Maxbin_out.019	*44_Firmicutes*	71.79	79.19	1.92	3731550	5663
S26_Maxbin_out.023	*153_Lachnospiraceae unclassified*	56.01	78.02	4.93	1682601	5883

S26_Maxbin_out.024	*172_Lachnospiraceae unclassified*	86.46	87.42	0.63	4743646	24342
S26_Maxbin_out.027	*38_Adlercreutzia spp.*	93.12	91.39	0.13	2037701	58430
S26_Maxbin_out.033	*187_Firmicutes unclassified*	97.55	97.93	0.6	3935476	171634
S26_Maxbin_out.039	*161_Lachnospiraceae unclassified*	87.10	96.23	2.29	2267064	42766
S26_Maxbin_out.067	*66_Candidatus stoquefichus*	80.98	94.07	3.17	2851301	77553
S27_Maxbin_out.002	*1_Akkermansia muciniphila*	100.58	97.99	0	2320414	151272
S27_Maxbin_out.005	*88_Ruminococcus spp.*	100.51	97.99	0	3232259	111944
S27_Maxbin_out.008	*65_Erysipelotrichaceae unclassified*	102.54	100	0	4302686	122472
S27_Maxbin_out.011	*96_Eubacteriaceae unclassified*	97.18	94.74	0	2848537	77017
S27_Maxbin_out.013	*171_Lachnospiraceae unclassified*	64.52	87.01	4.91	3208587	13095
S27_Maxbin_out.022	*167_Lachnospiraceae unclassified*	84.19	93.29	2.35	3014586	200878
S27_Maxbin_out.024	*22_Prevotellaceae unclassified*	98.77	99.43	0.63	3512636	97668
S27_Maxbin_out.032	*110_Clostridiales unclassified*	-24.29	75.86	22.41	4186281	62035
S27_Maxbin_out.044	*120_Firmicutes unclassified*	32.63	88.26	11.63	2176900	109805
S27_Maxbin_out.057	*165_Blautia spp.*	57.70	78.07	4.42	2825134	2914
S27_Maxbin_out.058	*179_Firmicutes unclassified*	50.13	92.43	8.89	3332402	19865
S28_Maxbin_out.007	*125_Firmicutes unclassified*	99.14	99.48	0.52	1998594	33355
S28_Maxbin_out.038	*23_Prevotellaceae unclassified*	92.37	93.12	0.63	3763038	62284
S28_Maxbin_out.060	*82_Clostridiaceae unclassified*	-26.36	84.06	22.61	5005740	6088
S28_Maxbin_out.068	*NA*	65.52	82.72	3.85	2347949	12334
S29_Maxbin_out.005	*52_Lactobacillus taiwanensis*	97.71	95.3	0.02	2640522	94532
S29_Maxbin_out.009	*12_Bacteroidetes unclassified*	96.64	97.29	0.61	2796579	34096
S29_Maxbin_out.011	*155_Lachnospiraceae unclassified*	95.64	99.35	1.27	4182792	188437
S29_Maxbin_out.018	*185_Dorea spp.*	44.51	76.78	7.36	1799726	8068
S29_Maxbin_out.030	*103_Firmicutes unclassified*	57.95	97.76	8.41	4047754	30660
S29_Maxbin_out.034	*108_Ruminococcaceae unclassified*	75.38	95.55	4.51	4512144	58299
S29_Maxbin_out.035	*131_Lachnospiraceae unclassified*	60.22	79.85	4.47	3065274	10845
S29_Maxbin_out.038	*156_Lachnospiraceae unclassified*	95.97	99.42	1.17	3095025	61928
S29_Maxbin_out.041	*135_Acetatifactor muris*	99.60	98.69	0.32	5082256	102428
S29_Maxbin_out.043	*33_Bifidobacterium pseudolongum*	80.76	78.19	0	2134156	139346
S29_Maxbin_out.065	*160_Firmicutes unclassified*	40.06	85.43	9.56	2465737	7264
S29_Maxbin_out.068	*72_Lachnospiraceae unclassified*	-46.46	76.18	24.92	3069406	8234
S3_Maxbin_out.020	*51_Lactobacillus taiwanensis*	-14.08	85.34	20.69	1318389	28774
S3_Maxbin_out.044	*7_Bacteroidaceae unclassified*	64.23	77.34	3.07	1922960	29863
S30_Maxbin_out.004	*175_Lachnospiraceae unclassified*	93.68	97.99	1.34	2932707	61488
S30_Maxbin_out.009	*97_Eubacteriaceae unclassified*	100.55	97.96	0	2643915	152196
S30_Maxbin_out.014	*142_Lachnoclostridium spp.*	100.24	98.86	0.26	1894487	154526
S30_Maxbin_out.017	*48_Lactobacillus reuteri*	101.60	99.15	0	1992142	80774
S30_Maxbin_out.022	*3_Alistipes spp.*	96.38	98.51	0.93	3593944	110783
S30_Maxbin_out.025	*71_Eubacterium spp.*	93.89	99.42	1.62	3985694	138088
S30_Maxbin_out.030	*86_Anaerotruncus spp.*	89.06	96.84	2.08	4642729	66051
S30_Maxbin_out.058	*98_Clostridiaceae unclassified*	7.23	91.07	17.45	2789930	6145
S30_Maxbin_out.072	*85_Firmicutes unclassified*	38.12	89.93	10.96	2278620	22255
S30_Maxbin_out.076	*73_Clostridium spp.*	58.38	86.21	6.04	2524434	5472

S30_Maxbin_out.092	*111_Oscillibacter spp.*	-22.52	97.78	24.53	5702437	13054
S31_Maxbin_out.010	*95_Firmicutes unclassified*	101.12	98.47	0	5914323	197095
S31_Maxbin_out.015	*114_Ruminococcaceae unclassified*	99.14	99.42	0.58	2787228	113104
S31_Maxbin_out.020	*13_Prevotellaceae unclassified*	99.34	98.66	0.34	3815279	58536
S31_Maxbin_out.024	*132_Dorea spp.*	95.16	92.83	0	3862158	46418
S31_Maxbin_out.036	*6_Tannerellaceae unclassified*	92.90	90.94	0.08	2335644	36619
S31_Maxbin_out.038	*174_Lachnospiraceae unclassified*	4.36	80.36	15.68	2159949	61755
S31_Maxbin_out.041	*126_Flavonifractor plautii*	19.63	77.99	12.08	1870733	11797
S32_Maxbin_out.018	*149_Firmicutes unclassfied*	98.51	98.45	0.48	3742971	83886
S32_Maxbin_out.019	*89_Ruminococcaceae unclassified*	97.22	98.56	0.77	4174706	104863
S32_Maxbin_out.022	*9_Bacteroidaceae unclassified*	89.79	87.93	0.09	2225989	41825
S32_Maxbin_out.023	*29_Sutterellaceae unclassified*	95.50	94.05	0.29	2631181	628974
S32_Maxbin_out.028	*2_Alistipes finegoldii*	96.49	97.32	0.67	2480651	109358
S32_Maxbin_out.032	*178_Firmicutes unclassified*	100.59	99.19	0.23	2179913	125452
S32_Maxbin_out.043	*166_Clostridium symbiosum*	83.32	92.13	2.25	1244121	76085
S33_Maxbin_out.031	*186_Dorea spp.*	36.41	78.85	8.94	4164924	10317
S33_Maxbin_out.044	*21_Prevotellaceae unclassified*	90.94	89.21	0.03	1845493	5638
S33_Maxbin_out.048	*83_Ruminococcaceae unclassified*	98.56	96.14	0	4141680	68133
S33_Maxbin_out.051	*99_Hungateiclostridiaceae*	84.22	98.94	3.42	2298848	58041
S33_Maxbin_out.079	*68_Eubacterium spp.*	62.69	92.21	6.39	3593793	16706
S34_Maxbin_out.006	*53_Lactobacillus johnsonii*	101.66	100	0.16	3287339	83441
S34_Maxbin_out.018	*140_Clostridium bolteae*	78.08	79.02	0.67	2037797	66954
S34_Maxbin_out.023	*112_Ruminococcaceae unclassified*	54.67	96.59	8.89	2998275	17582
S34_Maxbin_out.026	*124_Firmicutes unclassified*	21.01	82.01	12.75	2852720	53018
S35_Maxbin_out.006	*176_Eubacterium spp.*	89.43	97.13	2.01	4306440	50826
S35_Maxbin_out.009	*169_Lachnospiraceae unclassified*	36.73	80.04	9.16	3899188	96864
S35_Maxbin_out.010	*59_Clostridium innocuum*	102.23	100	0	2067123	29318
S35_Maxbin_out.014	*158_Eisenbergiella tayi*	69.85	85.2	3.51	2283171	4624
S35_Maxbin_out.017	*67_Peptostreptococcaceae*	88.47	88.84	0.6	2300757	184593
S35_Maxbin_out.020	*121_Firmicutes unclassified*	-3.71	79.7	17.4	1496252	2666
S35_Maxbin_out.025	*70_Firmicutes unclassified*	88.36	96.96	2.15	3644085	20044
S35_Maxbin_out.033	*57_Faecalibaculum rodentium*	85.58	83.21	0	2315146	55839
S36_Maxbin_out.019	*30_Sutterellaceae unclassified*	91.73	89.24	0	2379100	93799
S36_Maxbin_out.030	*34_Enorma spp.*	102.04	99.6	0	2137421	75607
S36_Maxbin_out.032	*79_Erysipelotrichaceae*	79.48	93.29	3.29	2352196	19834
S36_Maxbin_out.042	*118_Firmicutes unclassified*	21.87	77.97	11.69	2178044	3369
S4_Maxbin_out.013	*181_Ruminococcus torques*	95.81	97.18	0.81	3138762	225937
S4_Maxbin_out.032	*115_Ruminococcaceae unclassified*	93.74	97.78	1.29	3630966	66828
S4_Maxbin_out.034	*101_Anaerotruncus colihominis*	17.87	85.12	13.93	2411331	10444
S4_Maxbin_out.035	*162_Lachnospiraceae unclassified*	87.22	97.7	2.57	4080614	54006
S4_Maxbin_out.047	*10_Prevotellaceae unclassified*	78.28	99.33	4.7	4379299	80942
S4_Maxbin_out.052	*87_Anaerotruncus spp.*	94.31	94.83	0.57	4285011	45220
S4_Maxbin_out.056	*41_Firmicutes unclassfied*	98.83	96.69	0	1889246	18792

S4_Maxbin_out.065	*24_Proteobacteria unclassified*	54.66	90.15	7.5	2902693	10299
S4_Maxbin_out.067	*77_Firmicutes unclassified*	99.10	97.52	0.16	2821485	56359
S4_Maxbin_out.069	*133_Clostridium bolteae*	87.44	98.44	2.96	1937527	22951
S5_Maxbin_out.015	*20_Prevotellaceae unclassified*	97.06	99.31	0.94	3485196	79628
S5_Maxbin_out.018	*116_Clostridiales unclassified*	100.72	98.24	0	3497803	91534
S5_Maxbin_out.021	*129_Clostridium spp.*	99.39	96.97	0	2748640	70589
S5_Maxbin_out.028	*78_Firmicutes unclassified*	93.38	91.51	0.13	2092965	108771
S5_Maxbin_out.030	*150_Blautia spp.*	89.62	87.34	0	4396473	36176
S5_Maxbin_out.033	*94_Firmicutes unclassified*	33.65	86.49	11.07	2616849	11627
S6_Maxbin_out.001	*8_Bacteroides spp.*	93.01	94.35	0.88	2586927	22819
S6_Maxbin_out.027	*106_Oscillibacter spp.*	88.52	91.93	1.33	5818531	51705
S6_Maxbin_out.028	*50_Lactobacillus intestinalis*	75.03	92.6	4.03	4966874	42506
S6_Maxbin_out.036	*26_Azospirillum spp.*	85.99	95.98	2.49	4505108	83567
S7_Maxbin_out.003	*164_Lachnospiraceae unclassified*	91.91	97.32	1.57	2937561	74330
S7_Maxbin_out.017	*18_Muribaculum intestinale*	100.49	97.99	0	3243022	99074
S7_Maxbin_out.038	*84_Anaerotruncus colihominis*	99.80	98.35	0.21	6205463	101814
S7_Maxbin_out.046	*75_Firmicutes unclassified*	99.63	97.97	0.19	2859025	167161
S7_Maxbin_out.053	*170_Schaedlerella arabinosiphila*	-39.86	77.26	23.95	2965195	3781
S8_Maxbin_out.020	*159_Firmicutes unclassified*	80.02	98.85	4.31	7483420	276895
S8_Maxbin_out.048	*32_Rothia nasimurium*	100.34	99.37	0.32	5601687	141146
S8_Maxbin_out.064	*74_Anaerotruncus spp.*	67.67	78.95	2.63	2425216	5384
S9_Maxbin_out.006	*5_Parabacteroides goldsteinii*	92.68	90.11	0	1977927	137926
S9_Maxbin_out.013	*63_Candidatus stoquefichus*	102.58	100	0	3380293	144406
S9_Maxbin_out.020	*64_Erysipelotrichaceae| unclassified*	92.95	96.55	1.2	4384970	64558
S9_Maxbin_out.027	*182_Ruminococcus gnavus*	92.25	99.37	1.9	3478964	56687
S9_Maxbin_out.051	*76_Anaerotruncus colihominis*	51.78	97.7	9.77	4630218	102332

Supplementary Table 2. Accession number of query sequences used for BLAST

Pathway ID	Gene	Protein	Organism Genus	UniProt / NCBI accession	Reference
AK	*ackA*	Acetate Kinase	*Escherichia coli*	P0A6A3	(1)
			Enterococcus faecalis	Q833H0	(2)
			Bacteroides thetaiotaomicron	Q8A1G8	(3)
			Lachnoclostridium phytofermentans	A9KNV3	(4)
			Lactobacillus paracasei	Q03CP2	(5)
	ackA2	Acetate Kinase 2	*Clostridium perfringens*	Q8XJN2	(4)
BK	*buk*	Butyrate Kinase	*Clostridium perfringens*	P0C2D8	(6)
			Bacteroides vulgatus	BUK_BACV8	(6)
			Desulfovibrio salexigens	BUK_CLOBB	(6)
	ptb	Phosphate butyryltransferase	*Clostridium beijerinckii*	Q05624	(6)
BcoA	*RHOM_13820*	Butyryl: acetate CoA-transferase	*Roseburia hominis*	G2SYC0	(6)
			Anaerostipes caccae	B0MC58	(6)
McoA	*mmdA*	Methylmalonyl-CoA decarboxylase alpha subunit	*Veillonella parvula*	ZP_06259922.1	(6)
	MmdB_2	putative methylmalonyl-CoA decarboxylase, beta subunit	*Bacteroides thetaiotaomicron*	A0A174QKQ8	(3,7)
	scpB	Methylmalonyl-CoA decarboxylase	*Escherichia coli*	P52045	(8)
	mcoA	Methylmalonyl-CoA carboxytransferase	*Akkermansia muciniphila*	B2UP73	(9,10)
Lacdh	*ldh*	L-lactate Dehydrogenase	*Limosilactobacillus reuteri*	Q8GMJ0	(11)
			Bifidobacterium longum subsp. Longum	E8ME30	(12)
			Escherichia coli	P33232	(13,14)
			Desulfovibrio vulgaris subsp. vulgaris	A1VG02	(15)
	ldhA	D-lactate dehydrogenase	*Lactobacillus delbrueckii subsp. bulgaricus*	P26297	(16)
LacRd	*fucO*	Lactaldehyde Reductase	*Escherichia coli*	P0A9S1	(14)
LacCoRd	*lcdA*	Lactoyl-CoA Dehydratase subunit alpha	*Anaerotignum propionicum*	G3KIM4	(6)
frdA	*frdB*	Fumarate reductase transmembrane cytochrome b subunit	*Bacteroides fragilis*	Q5L7E7	(6)
	Amuc_0986	fumarate reductase	*Akkermansia muciniphila*	ACD04815.1	(10,17)
	frdC	Fumarate reductase subunit C	*Escherichia coli*	P0A8Q0	(18)
pduCDE	*pduC*	Propanediol dehydratase large subunit	*Salmonella typhimurium*	P37450	(19,20)

	pduD	Propanediol Dehydratase medium subunit	*Salmonella typhimurium*	O31041	(19,20)
	pduE	Propanediol dehydratase small subunit	*Salmonella typhimurium*	O31042	(19,20)
CbiM	*Amuc_1200*	Cobalt transporter Protein CbiM	*Akkermansia muciniphila*	WP_012420715.1	(10)
	btuB	Vitamin B12 transporter BtuB	*Escherichia coli*	P06129	(21)
	btuC	Vitamin B12 import system permease protein BtuC	*Escherichia coli*	P06609	(21)
	btuF	Vitamin B12 import system permease protein BtuF	*Escherichia coli*	P37028	(21)
	btuD	Vitamin B12 import system permease protein BtuD	*Escherichia coli*	P06611	(21)
	CbiM	Cobalt transport protein CbiM	*Lachnoclostridium phytofermentans*	A9KP98	(21)
			Clostridium cellulovorans	D9SNZ5	(21)
			Desulfotomaculum reducens	A4J832	(21)
BbgI	*BbgI*	Beta-galactosidase BbgI	*Bifidobacterium bifidum*	Q0ZI53	(22)
LacZ	*LacZ*	Beta-galactosidase LacZ	*Lactobacillus acidophilus*	A4K5H9	(23)
LacA	*LacA*	Beta-galactosidase LacA	*Lactobacillus acidophilus*	C6H178	(24,25)
Lacdeb	*Lacde*	Beta-galactosidase Lacde	*Lactobacillus delbrueckii subsp. Bulgaricus*	D4QFE8	(23,24)
LacM	*LacM*	Beta-galactosidase LacM	*Leuconostoc lactis*	Q02604	(24)

Supplementary Table 3. ANOSIM of cecal microbiota based on weighted UniFrac distance matrix

Category	Group 1	Group 2	Sample size	R	*p*-value	q-value
All	Dams	Offspring-W18	66	0.197	0.004	0.004
	Dams	Offspring-W3	52	0.388	0.001	0.002
	Offspring-W18	Offspring-W3	84	0.584	0.001	0.002
Dam	APM	WTR	11	-0.077	0.764	0.764
	APM	STV	12	0.119	0.144	0.216
	WTR	STV	11	0.114	0.119	0.216
Offspring-W3	APM	WTR	23	0.186	0.02	0.041
	APM	STV	23	0.125	0.041	0.041
	WTR	STV	24	0.124	0.028	0.041
Offspring-W18	APM	WTR	34	-0.036	0.817	0.817
	APM	STV	31	-0.017	0.592	0.817
	WTR	STV	33	-0.015	0.596	0.817

APM, aspartame; STV, stevia; WTR, water control; W, week.

Supplementary Table 4. The 50 most abundant 16S rRNA gene amplicon sequence variants (ASVs) in cecal microbiota of dams and offspring at 3 weeks and 18 weeks of age. Data are represented as mean ± SEM. Abundances in the same row are significantly different (p < 0.05) when they are not labeled with a common superscript. W3, week 3; W18, week 18; APM, aspartame; STV, stevia; WTR, water control; *ND*, not detected.

ASV ID	Taxon	Confidence	WTR-Dam	APM-Dam	STV-Dam	WTR-W3	APM-W3	STV-W3	WTR-W18	APM-W18	STV-W18
ASV1	*Bacteroides spp.*	1.00	1.05±0.66	0.25±0.08	0.6±0.22	0.57±0.56	0.26±0.18	1.61±0.97	0.15±0.04	0.09±0.05	0.32±0.09
ASV2	*Uncultured Bacteroidetes*	0.75	0.9±0.58	0.28±0.11	0.48±0.18	0.53±0.51	0.4±0.29	0.65±0.35	0.12±0.04	0.09±0.05	0.24±0.1
ASV3	*Uncultured Bacteroidetes*	0.75	0.68±0.44	0.32±0.16	0.39±0.14	0.4±0.39	0.45±0.36	0.85±0.46	0.1±0.04	0.1±0.05	0.24±0.08
ASV4	*Uncultured Muribaculaceae*	0.95	0.13±0.06	0.1±0.05	0.15±0.01	0.89±0.17	0.64±0.21	0.57±0.1	0.24±0.05	0.19±0.05	0.22±0.06
ASV5	*Uncultured Bacteroidetes*	0.87	***0.98±0.43***^{AB}	***1.01±0.21***^{B}	***2.46±0.61***^{A}	1.13±0.37	0.91±0.23	1.35±0.37	0.4±0.12	0.5±0.29	0.48±0.12
ASV6	*Uncultured Bacteroidetes*	0.81	0.87±0.4	0.88±0.28	2.2±0.55	0.97±0.31	0.8±0.2	1.16±0.31	0.38±0.11	0.45±0.28	0.42±0.11
ASV7	*Uncultured Bacteroidetes*	0.87	9.99±2.87	9.55±1.54	11.85±2.22	5.34±1.64	7.84±1.37	6.88±1.06	10.37±0.91	11.06±1.04	9.23±0.79
ASV8	*Uncultured Muribaculaceae*	0.96	0.11±0.04	0.16±0.06	0.12±0.04	0.82±0.24	0.5±0.11	0.71±0.1	0.1±0.03	0.12±0.04	0.15±0.03
ASV9	*Uncultured Muribaculaceae*	0.97	0.21±0.07	0.35±0.09	0.44±0.05	1.18±0.25	0.62±0.1	0.66±0.1	0.38±0.05	0.32±0.04	0.34±0.05
ASV10	*Uncultured Bacteroidetes*	0.79	0.97±0.38	2.19±0.59	3.6±0.69	***0.24±0.09***^{B}	***2.52±0.43***^{A}	***2.26±0.34***^{A}	***0.83±0.25***^{B}	***3.0±0.46***^{A}	***2.2±0.26***^{A}
ASV11	*Alloprevotella spp.*	0.99	0.01±0.26	1.61±1.6	ND	ND	ND	ND	4.09±1.16	3.86±0.96	5.34±1.44
ASV12	*Alistipes spp.*	1.00	0.17±0.09	0.07±0.02	0.07±0.02	1.71±1.1	0.76±0.41	1.76±0.55	0.04±0.01	0.04±0.02	0.08±0.02
ASV13	*Parabacteroides goldsteinii*	0.96	1.28±0.4	1.12±0.26	1.58±0.59	6.51±0.76	6.38±1.1	5.89±1.07	0.6±0.09	0.55±0.12	0.71±0.12
ASV14	*Limosilactobacillus reuteri*	1.00	0.57±0.35	0.14±0.09	0.21±0.14	***2.21±0.39***^{A}	***1.03±0.22***^{B}	***1.84±0.15***^{A}	0.25±0.07	0.32±0.1	0.4±0.11
ASV15	*Ligilactobacillus spp.*	1.00	ND	ND	ND	***1.54±0.35***^{A}	***0.66±0.27***^{B}	***0.44±0.1***^{B}	ND	ND	ND
ASV16	*Lactobacillus intestinalis*	1.00	0.45±0.34	0.15±0.12	0.15±0.08	0.63±0.24	0.44±0.26	0.44±0.11	0.16±0.06	0.22±0.08	0.28±0.1
ASV17	*Lactobacillus johnsonii*	1.00	4.74±2.8	3.06±1.3	3.25±1.85	***5.95±1.51***^{B}	***4.85±1.54***^{B}	***10.99±1.24***^{A}	***2.66±0.68***^{B}	***3.26±0.89***^{AB}	***5.68±1.27***^{A}
ASV18	*Ligilactobacillus spp.*	1.00	0.9±0.27	0.49±0.16	0.82±0.3	***7.95±1.76***^{A}	***3.84±1.61***^{AB}	***2.42±0.55***^{B}	3.4±0.74	2.01±0.41	1.56±0.27
ASV19	*Lactobacillus taiwanensis*	1.00	0.02±0.01	0.01±0.01	0.68±0.65	1.75±0.94	2.05±1.35	0.11±0.07	0.02±0.02	0.01±0.01	0.01±0.01
ASV20	*Limosilactobacillus reuteri*	1.00	1.27±0.88	0.26±0.16	0.6±0.27	***4.61±0.81***^{A}	***2.13±0.46***^{B}	***3.83±0.3***^{A}	0.56±0.15	0.7±0.19	0.86±0.19
ASV21	*Clostridium sensu stricto 1*	1.00	1.77±0.77	1.19±0.2	2.12±0.49	0.09±0.02	0.25±0.07	0.26±0.12	4.33±0.75	3.44±1.07	5.16±1.53
ASV22	*Uncultured Lachnospiraceae*	1.00	5.58±4.18	4.18±2.17	3.14±2.99	ND	0.37±0.22	0.04±0.02	1.86±0.99	0.1±0.06	0.94±0.51
ASV23	*Uncultured Lachnospiraceae*	1.00	0.81±0.64	0.02±0.01	0.09±0.04	1.91±0.61	1.62±0.47	1.43±0.35	0.01±0.01	0.02±0.02	0.25±0.16
ASV24	*Uncultured Lachnospiraceae*	1.00	0.69±0.27	1.26±0.21	0.67±0.32	0.44±0.12	0.47±0.15	0.49±0.11	0.62±0.17	0.34±0.09	0.25±0.07
ASV25	*Uncultured Firmicutes*	0.88	0.7±0.54	3.08±0.64	1.15±0.9	ND	0.17±0.16	0.01±0.01	0.54±0.18	1.26±0.44	0.62±0.25
ASV26	*Blautia spp.*	1.00	0.23±0.11	0.02±0.02	0.1±0.04	***2.05±0.33***^{A}	***1.2±0.52***^{AB}	***0.78±0.23***^{B}	ND	0.01±0.01	ND
ASV27	*Blautia spp.*	1.00	0.91±0.28	1.55±0.61	0.83±0.42	ND	0.1±0.06	0.03±0.02	0.99±0.19	0.94±0.25	1.02±0.22
ASV28	*Blautia spp.*	0.97	1.33±1.11	0.02±0.02	0.06±0.04	3.24±1.21	1.51±0.9	4.17±1.54	0.01±0.01	0.03±0.02	0.14±0.11
ASV29	*Blautia spp.*	1.00	2.75±1.16	7.39±2.25	4.34±2.31	0.16±0.09	0.87±0.38	0.67±0.29	0.67±0.18	1.57±0.85	0.58±0.14
ASV30	*Eisenbergiella spp.*	1.00	ND	ND	ND	2.08±0.51	0.71±0.3	0.88±0.18	ND	ND	ND
ASV31	*Eisenbergiella spp.*	0.99	0.03±0.03	ND	ND	1.09±0.51	0.45±0.32	0.71±0.38	ND	ND	ND
ASV32	*Hungatella spp.*	0.99	0.02±0.01	ND	ND	***3.92±1.2***^{A}	***0.44±0.2***^{B}	***2.34±1.1***^{A}	ND	ND	ND
ASV33	*Uncultured Lachnoclostridium*	0.98	0.78±0.65	0.02±0.01	0.09±0.08	2.98±0.72	1.07±0.33	2.1±0.46	ND	ND	ND
ASV34	*Uncultured Firmicutes*	0.83	ND	ND	ND	ND	ND	ND	1.99±0.67	1.11±0.49	1.48±0.9
ASV35	*Uncultured Firmicutes*	0.83	ND.02	0.17±0.14	ND	ND	ND	ND	4.75±0.89	5.85±1.05	5.76±1.34
ASV36	*Uncultured Firmicutes*	0.86	ND	1.39±0.6	ND	ND	0.04±0.02	ND	0.55±0.24	1.61±0.36	1.47±0.35
ASV37	*Uncultured Firmicutes*	0.87	1.16±0.9	0.44±0.28	1.54±0.81	ND	0.01±0.01	0.19±0.19	2.18±0.56	0.34±0.34	0.33±0.3

ASV38	*Uncultured Peptococcaceae*	0.99	0.54±0.21	0.56±0.06	0.62±0.09	0.12±0.03	0.41±0.1	0.27±0.06	0.69±0.06	0.62±0.08	0.58±0.09
ASV39	*Romboutsia spp.*	0.92	4.84±1.64	3.72±0.8	3.57±0.51	0.48±0.08	1.08±0.26	0.78±0.24	1.82±0.28	1.66±0.31	1.39±0.27
ASV40	*Romboutsia spp.*	0.92	4.65±1.46	4.45±0.77	4.51±0.55	0.53±0.09	1.39±0.33	1.11±0.32	1.56±0.22	1.33±0.3	1.01±0.2
ASV41	*Uncultured Firmicutes*	0.80	1.92±0.66	0.47±0.29	1.64±0.78	0.45±0.22	0.76±0.28	0.44±0.21	0.64±0.19	0.42±0.26	0.38±0.13
ASV42	*Uncultured Firmicutes*	0.80	0.83±0.3	0.77±0.08	1.1±0.24	0.16±0.13	0.49±0.23	0.18±0.1	0.83±0.11	1.22±0.17	1.18±0.14
ASV43	*Uncultured Firmicutes*	0.86	1.42±1.06	3.27±0.92	2.17±0.99	0.05±0.03	0.05±0.04	0.58±0.35	3.14±0.67	2.17±0.48	2.39±0.62
ASV44	*Uncultured Firmicutes*	0.74	ND	0.02±0.02	1.55±1.55	ND	ND	ND	4.94±0.84	5.32±1.17	4.53±0.95
ASV45	*Clostridium innocuum group*	1.00	0.1±0.04	0.04±0.02	0.09±0.03	5.3±0.86	3.09±0.99	5.85±1.51	0.01±0.01	ND	0.01±0.01
ASV46	*Uncultured Erysipelatoclostridium*	1.00	0.1±0.06	0.02±0.02	0.07±0.07	1.3±0.3	0.67±0.15	0.97±0.36	ND	0.01±0.01	0.01±0.01
ASV47	*Faecalitalea spp.*	1.00	0.14±0.12	ND	0.01±0.01	***1.25±0.4^{A}***	***0.4±0.37^{B}***	***0.85±0.38^{AB}***	0.04±0.03	0.02±0.01	0.14±0.1
ASV48	*Uncultured Firmicutes*	0.75	0.15±0.05	0.14±0.03	0.47±0.23	0.02±0.01	0.04±0.01	0.04±0.02	1.26±0.24	1.3±0.51	1.98±0.84
ASV49	*Escherichia-Shigella*	1.00	0.08±0.05	0.04±0.03	0.04±0.02	2.6±0.8	1.19±0.46	2.24±1.31	0.1±0.03	0.22±0.07	0.21±0.09
ASV50	*Akkermansia muciniphila*	1.00	***14.61±2.63^{A}***	***9.96±2.9^{AB}***	***6.1±1.52^{B}***	***9.02±1.76^{B}***	***19.4±3.51^{A}***	***10.4±1.86^{B}***	6.69±1.17	6.93±1.23	5.22±1.27

1.2 Supplementary Figure

Supplementary Figure 1. Metabolic traits of genomes influenced by maternal consumption of aspartame and stevia. The metabolic traits of each genome were predicted by the presence / absence of the key enzyme(s) involving in SCFA metabolic pathways. Enzymes are selected to represent different pathways as follows: **Acetate**: acetate kinase (AK) for acetate kinase pathway; **Propionate**: fumarate reductase (frdA) and methylmalonyl-CoA decarboxylase (McoA) for succinate decarboxylation pathway; lactaldehyde reductase (LacRd) and lactoyl-CoA dehydratase (LacCoRd) for acrylate pathway; propanediol dehydratase (pduCDE) for propanediol pathway; **Butyrate:** butyrate kinase (BK) for butyrate kinase and Butyryl:acetate CoA-transferase (BCoA); **Lactate**: L-lactate dehydrogenase (Lacdh) for lactate production from pyruvate; **Cobalamin (Vitamin B12):** cobalt transporter protein (CbiM) for cobalamin production. The accession number of query sequences and references to the biochemical characterization of the enzymes is provided in Table S1.

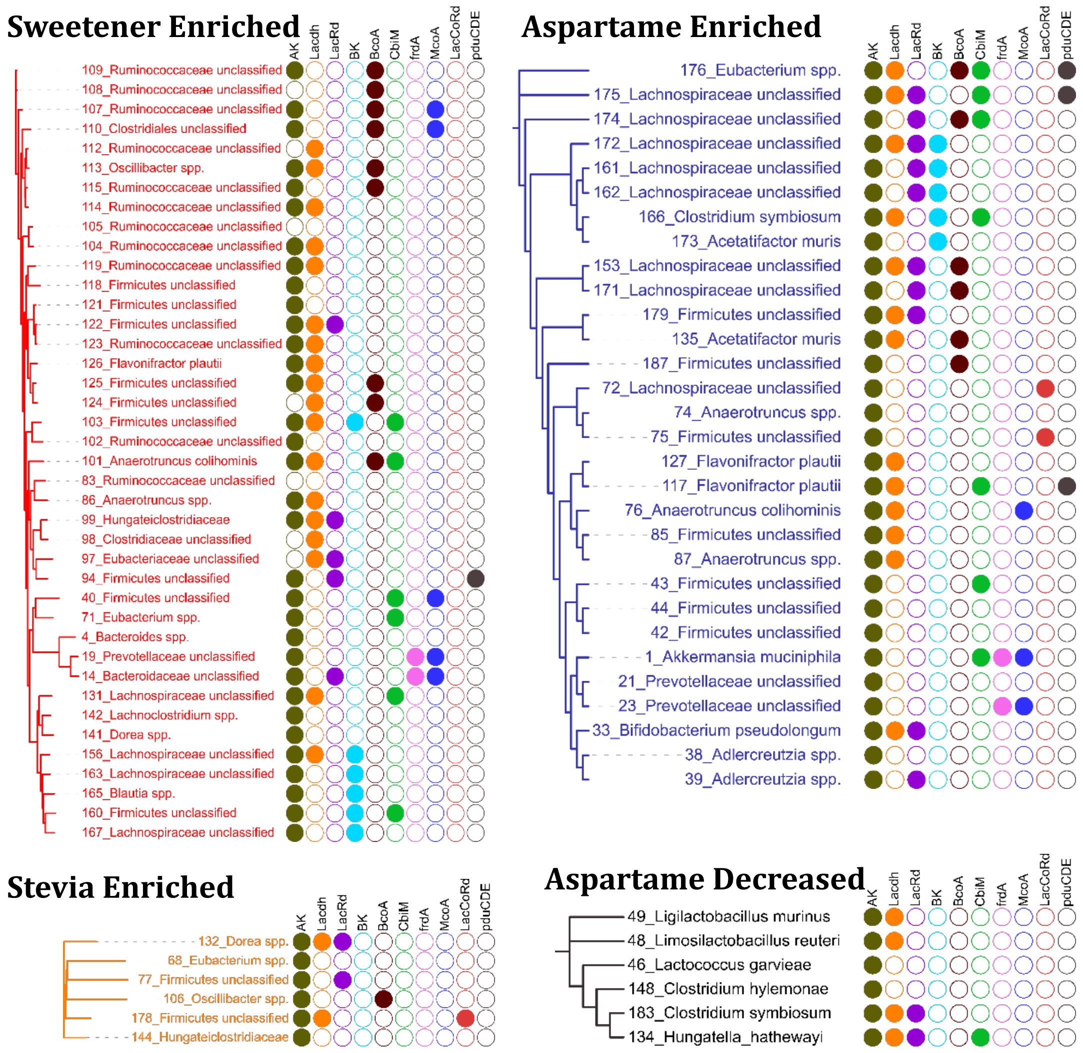

AK Acetate Kinase **BK** Butyrate Kinase **frdA** Fumarate Reductase **pduCDE** Propanediol Dehydratase
Lacdh L-lactate Dehydrogenase **LacRd** Lactaldehyde Reductase **LacCoRd** Lactoyl-CoA Dehydratase
BCoA Butyryl: acetate CoA-transferase **CbiM** Cobalt transporter **McoA** Methylmalonyl-CoA

2 References

1. Dittrich CR, Bennett GN, San KY. Characterization of the acetate-producing pathways in *Escherichia coli* . *Biotechnol Prog* (2005) 21(4):1062–7. doi:10.1021/bp050073s

2. Louis P, Duncan SH, McCrae SI, Millar J, Jackson MS, Flint HJ. Restricted distribution of the butyrate kinase pathway among butyrate-producing bacteria from the human colon. *J Bacteriol* (2004) 186(7):2099–106. doi:10.1128/JB.186.7.2099-2106.2004

3. Ryan D, Jenniches L, Reichardt S, Barquist L, Westermann AJ. A high-resolution transcriptome map identifies small RNA regulation of metabolism in the gut microbe Bacteroides thetaiotaomicron. *Nat Commun* (2020) 11(1):1–16. doi:10.1038/s41467-020-17348-5

4. Yu HY, Meade A, Liu SJ. Phylogeny of *Clostridium* spp. based on conservative genes and comparisons with other trees. *Microbiology* (2019) 88(4):469–78. doi:10.1134/S002626171904012X

5. Goh YJ, Zhang C, Benson AK, Schlegel V, Lee JH, Hutkins RW. Identification of a putative operon involved in fructooligosaccharide utilization by *Lactobacillus paracasei*. *Appl Environ Microbiol* (2006) 72(12):7518–30. doi:10.1128/AEM.00877-06

6. Reichardt N, Duncan SH, Young P, Belenguer A, McWilliam Leitch C, Scott KP, et al. Phylogenetic distribution of three pathways for propionate production within the human gut microbiota. *ISME J* (2014) 8(6):1323–35. doi:10.1038/ismej.2014.14

7. Ikeyama N, Murakami T, Toyoda A, Mori H, Iino T, Ohkuma M, et al. Microbial interaction between the succinate-utilizing bacterium *Phascolarctobacterium faecium* and the gut commensal *Bacteroides thetaiotaomicron*. *Microbiologyopen* (2020) 9(10). doi:10.1002/mbo3.1111

8. Kandasamy V, Vaidyanathan H, Djurdjevic I, Jayamani E, Ramachandran KB, Buckel W, et al. Engineering *Escherichia coli* with acrylate pathway genes for propionic acid synthesis and its impact on mixed-acid fermentation. *Appl Microbiol Biotechnol* (2013) 97(3):1191–200. doi:10.1007/s00253-012-4274-y

9. Crost EH, Le Gall G, Laverde-Gomez JA, Mukhopadhya I, Flint HJ, Juge N. Mechanistic insights into the cross-feeding of Ruminococcus gnavus and Ruminococcus bromii on host and dietary carbohydrates. *Front Microbiol* (2018) 9(11):2558. doi:10.3389/fmicb.2018.02558

10. Xing J, Li X, Sun Y, Zhao J, Miao S, Xiong Q, et al. Comparative genomic and functional analysis of *Akkermansia muciniphila* and closely related species. *Genes and Genomics* (2019) 41(11):1253–64. doi:10.1007/s13258-019-00855-1

11. Alayande KA, Aiyegoro OA, Nengwekhulu TM, Katata-Seru L, Ateba CN. Integrated genome-based probiotic relevance and safety evaluation of Lactobacillus reuteri PNW1. *PLoS One* (2020) 15(7):e0235873. doi:10.1371/journal.pone.0235873

12. Takashi M, So I, Hiroshi S, Haruhiko M, Takahisa O. Sequence and characteristics of the *Bifidobacterium longum* gene encoding l-lactate dehydrogenase and the primary structure of the enzyme: a new feature of the allosteric site. *Gene* (1989) 85(1):161–8. doi:10.1016/0378-1119(89)90476-9

13. Bunch PK, Mat-Jan F, Lee N, Clark DP. The *IdhA* gene encoding the fermentative lactate dehydrogenase of *Escherichia coli*. *Microbiology* (1997) 143(1):187–95. doi:10.1099/00221287-143-1-187

14. Cocks GT, Aguilar J, Lin ECC. Evolution of l-1,2-propanediol catabolism in *Escherichia coli* by recruitment of enzymes for l-fucose and l-lactate metabolism. *J Bacteriol* (1974) 118(1)

15. Vita N, Valette O, Brasseur G, Lignon S, Denis Y, Ansaldi M, et al. The primary pathway for lactate oxidation in Desulfovibrio vulgaris. *Front Microbiol* (2015) 6(6):606. doi:10.3389/fmicb.2015.00606

16. Bernard N, Ferain T, Garmyn D, Hols P, Delcour J. Cloning of the D-lactate dehydrogenase gene from *Lactobacillus delbrueckii* subsp. *bulgaricus* by complementation in *Escherichia coli*. *FEBS Lett* (1991) 290(1–2):61–4. doi:10.1016/0014-5793(91)81226-X

17. Chia LW, Hornung BVH, Aalvink S, Schaap PJ, de Vos WM, Knol J, et al. Deciphering the trophic interaction between *Akkermansia muciniphila* and the butyrogenic gut commensal *Anaerostipes caccae* using a metatranscriptomic approach. *Int J Gen Mol Microbiol* (2018) 111(6):859–73. doi:10.1007/s10482-018-1040-x

18. Iverson TM, Luna-Chavez C, Cecchini G, Rees DC. Structure of the *Escherichia coli* fumarate reductase respiratory complex. *Science* (1999) 284(5422):1961–6. doi:10.1126/science.284.5422.1961

19. Altaras NE, Cameron DC. Metabolic engineering of a 1,2-propanediol pathway in *Escherichia coli*. *Appl Environ Microbiol* (1999) 65(3):1180–5. doi:10.1128/aem.65.3.1180-1185.1999

20. Walter D, Ailion M, Roth J. Genetic characterization of the pdu operon: use of 1,2-propanediol in *Salmonella typhimurium*. *J Bacteriol* (1997) 179(4):1013–22

21. Raux E, Schubert HL, Warren MJ. Biosynthesis of cobalamin (vitamin B12): A bacterial conundrum. *Cell Mol Life Sci* (2000) 57(13):1880–93. doi:10.1007/PL00000670

22. Goulas TK, Goulas AK, Tzortzis G, Gibson GR. Molecular cloning and comparative analysis of four β-galactosidase genes from *Bifidobacterium bifidum* NCIMB41171. *Appl Microbiol Biotechnol* (2007) 76(6):1365–72. doi:10.1007/s00253-007-1099-1

23. Gänzle MG, Follador R. Metabolism of oligosaccharides and starch in lactobacilli: A review. *Front Microbiol* (2012) 3(9):340. doi:10.3389/fmicb.2012.00340

24. Schwab C, Sørensen KI, Gänzle MG. Heterologous expression of glycoside hydrolase family 2 and 42 β-galactosidases of lactic acid bacteria in *Lactococcus lactis*. *Syst Appl Microbiol* (2010) 33(6):300–7. doi:10.1016/j.syapm.2010.07.002

25. Wang W, Hu H, Zijlstra RT, Zheng J, Gänzle MG. Metagenomic reconstructions of gut microbial metabolism in weanling pigs. *Microbiome* (2019) 7(1):48. doi:DOI: 10.1186/s40168-019-0662-1
